# Facile Solvothermal Synthesis of Hollow BiOBr Submicrospheres with Enhanced Visible-Light-Responsive Photocatalytic Performance

**DOI:** 10.1155/2020/3058621

**Published:** 2020-03-09

**Authors:** Linrui Hou, Yawei Niu, Fan Yang, Fengyue Ge, Changzhou Yuan

**Affiliations:** School of Material Science and Engineering, University of Jinan, Jinan 250022, China

## Abstract

In this work, hierarchical hollow BiOBr submicrospheres (HBSMs) were successfully prepared *via* a facile yet efficient solvothermal strategy. Remarkable effects of solvents upon the crystallinities, morphologies, and microstructures of the BiOBr products were systematically investigated, which revealed that the glycerol/isopropanol volumetric ratio played a significant role in the formation of hollow architecture. Accordingly, the underlying formation mechanism of the hollow submicrospheres was tentatively put forward here. Furthermore, the photocatalytic activities of the resulting HBSMs were evaluated in detail with photocatalytic degradation of the organic methyl orange under visible light irradiation. Encouragingly, the as-obtained HBSMs with striking recyclability demonstrated excellent visible-light-responsive photocatalytic performance, which benefits from their large surface area, effective visible light absorption, and unique hollow feature, highlighting their promising commercial application in waste water treatment.

## 1. Introduction

In recent years, semiconductor photocatalysis has gradually been recognized as a promising approach to effectively solve the ever-increasing energy shortage and environmental pollution [1, 2]. Among various photocatalytic materials, BiOBr is of particular interest thanks to its stability, suitable bang gap, visible-light-response performance, and desirable photocatalytic activities [3–5]. Up till now, numerous pioneer researches have proved that the photocatalytic activities of a semiconductor are hugely dependent upon its size, configuration, and shape [6, 7]. In this context, the specific microstructures of photocatalysts have been tuned finely to improve their photocatalytic activities [8–10]. Furthermore, the BiOBr-based materials with various shapes including nanoplates, nanosheets (NSs), microspheres, and nanoflakes have been well exploited for photocatalytic applications [11–15]. In particular, the photocatalysts of hollow interiors always display the inimitable advantages in comparison with their solid counterparts, benefiting from the enhanced light-harvesting capacities through the light multiple reflection within the interior cavity, effective separation of photogenerated electron-hole pairs, and larger specific surface area (SSA) [16–19].

The template-assisted approaches constitute a constant focus of researches due to their superiorities in constructing hollow architectures towards a broad range of research fields and industrial processes [17, 20, 21]. For instance, Han et al. prepared the NiCO_2_S_4_/Co_9_S_8_ hollow spheres *via* the solvothermal method coupled with anion exchange process by using carbon spheres as the sacrificial template [22]. Jia and coworkers synthesized the luminescent Y_2_O_3_ hollow spheres with colloidal melamine formaldehyde template [23]. Also, our group previously synthesized mesoporous hollow NiCo_2_O_4_ submicrospheres towards supercapacitors by applying silica spheres as hard templates [24]. Unfortunately, the hard template-engaged methodologies inevitably suffer from the time-/energy-consuming issues, due to the involved multistep processes, and partial structural collapse with the removal of templates, which thereby stimulates the interest in the surfactant-engineered or even template-free fabrication of hollow architectures [25–27]. As for the BiOBr, Zhang et al. synthesized NSs-assembled BiOBr microspheres with non-close-packed structure *via* a microwave-assisted solvothermal route, and they exhibited good adsorptive capacity and excellent photocatalytic activity for organic dye [28]. Xia and coworkers prepared the porous hollow BiOBr spheres with a unique ionic liquid of 1-hexadecyl-3-methylimidazolium bromide [29]. Although enormous advances have been made for the smart synthesis of hollow BiOBr photocatalysts, there still remains huge research space to further boost their photocatalytic efficiency and expand their applications. Thus, it is highly desirable to develop a simple, green, and efficient avenue to prepare hollow BiOBr with high photocatalytic degradation performance for organic pollutants.

With the overviews above in mind, herein, we first scalably prepared hollow BiOBr submicrospheres (HBSMs) by using a simple solvothermal method, where the di-n-decyldimethylammonium bromide (DDAB) acts as Br source and the mixed glycerol (GC)/isopropanol (IP) as the solvent. The key role of the DDAB in the formation of the HBSMs and effects of various solvent systems upon the crystal phases, morphologies, and photocatalytic activities of the obtained BiOBr samples were systematically investigated. The photocatalytic activities and recyclability of the resulting HBSMs were examined by using methylene orange (MO) as the target pollutant under the Xenon arc lamp irradiation. Besides, the underlying formation mechanism of the HBSMs as well as their photocatalytic degradation mechanism for the dye MO was tentatively shed light upon here.

## 2. Experimental

### 2.1. Materials

Bi(NO_3_)_3_·5H_2_O, DDAB, Degussa P25 TiO_2_ (P25), GC, and IP of analytical grade were all purchased from Sinopharm Chemical Reagent Co., Ltd, and used as received without further purification.

### 2.2. Preparation of the BiOBr Samples

**Figure 1:**
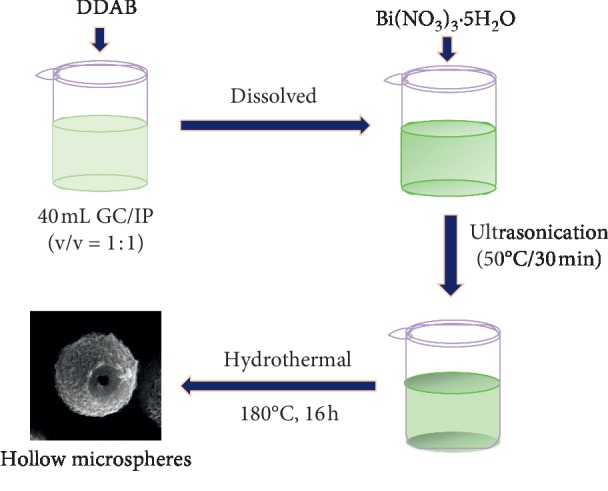
Synthesis flowchart for HBSMs.

Hollow BiOBr submicrospheres (HBSMs) were prepared *via* a facile solvothermal method, and corresponding synthetic route was presented in Figure 1. In a typical synthesis of the HBSMs, 0.488 g of DDAB was dissolved into a solution containing 20 mL of GC and 20 mL of IP in an ultrasonic bath. Afterwards, 0.485 g of Bi(NO_3_)_3_·5H_2_O was added to the above solution, and then the mixed solution was kept at 50°C for 30 min under ultrasonication. Subsequently, the mixture was transferred into a Teflon-lined autoclave (50 mL) and kept at 180°C for 16 h. After being cooled to room temperature naturally, the resulting precipitate was collected by centrifugation, washed, and further dried at 80°C for 6 h before further characterizations. For comparison, another two BiOBr samples were further obtained with similar procedure just by using the GC or IP as the sole solvent, and the corresponding products were denoted as BiOBr-GC and BiOBr-IP for convenience. The detailed synthetic parameters were shown in Table 1.

### 2.3. Materials Characterizations

The crystalline phases of samples were determined on powder X-ray diffractometer (XRD, Rigaku Ultima IV, Japan). Typical morphologies and microstructures of the samples were characterized by using field-emission scanning electron microscopy (FESEM, JEOL-6300F), transmission electron microscopy (TEM), high-resolution TEM (HRTEM), scanning TEM (STEM), and selected area electron diffraction (SAED) (JEOL JEM 2100 system). The Brunauer-Emmett-Teller (BET) SSA of the samples was calculated from N_2_ adsorption-desorption isotherms measured on a surface area analyzer (TriStar II 3020) at liquid nitrogen temperature. UV-vis diffuse-reflectance spectra were performed to determine the band gap energy (*E*_*g*_) of the photocatalysts by using a Hitachi U-3010 spectrophotometer. The total organic carbon analysis (TOC) was obtained on Shimadzu TOC-VCPN. X-ray photoelectron spectroscopy (XPS) was recorded by a PHI 5000 X-ray photoelectron spectrometer equipped with an Al K*α* (∼1486.6 eV) radiation source, and the spectra were well fitted using the XPSPEAK41 software.

### 2.4. Photocatalytic Evaluation

Photocatalytic activities of the samples were evaluated by photocatalytic degradation of the MO under visible light irradiation. A 350 W Xe arc lamp with a cutoff filter (*λ* > 400 nm) was furnished as the visible light source in the photoreaction system. Typically, 0.3 g of the photocatalyst was dispersed in 100 mL of the MO aqueous solution with an initial concentration of 2 × 10^−5^ M in a quartz reactor. Prior to illumination, the suspension solution was stirred magnetically in dark for 60 min to ensure an adsorption/desorption equilibrium. Afterwards, the solution was exposed to visible light irradiation under magnetic stirring. At given time intervals, an aliquot of 3 mL of suspension solution was drawn and then centrifuged to remove the particles (4000 rpm, 10 min). The optical absorption spectra for the supernatant solution were recorded by a double-beam Shimadzu UV-3600 spectrophotometer (Japan).

### 2.5. Active Species Trapping Experiment

To determine the active species produced in the photocatalytic process, such as hydroxyl radical (·OH), superoxide radical (·O_2_^−^), and hole (h^+^), the isopropyl alcohol (IPA), benzoquinone (BQ), and ethylenediaminetetraacetate (EDTA-2Na) with concentration of 1 mM are separately introduced into the photocatalytic reaction solution [30, 31].

## 3. Results and Discussion

### 3.1. Structural and Physicochemical Characterizations

In this work, a facile solvothermal methodology was devised to prepare the HBSMs with a mixed solvent of GC and IP (1 : 1, v : v). As is well known, the solvents generally render certain effects on the crystal growth and even phase composition of a semiconductor over the solvothermal process [32–34]. To this end, the GC or IP was also adopted as a single solvent to synthesize BiOBr samples, as depicted in Table 1.

**Figure 2:**
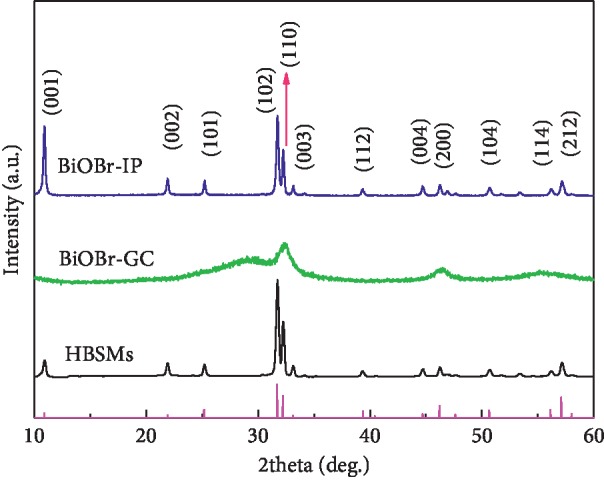
XRD patterns of the resultant HBSMs, BiOBr-GC, and BiOBr-IP samples. The magenta vertical lines for the standard spectrum of the BiOBr (JCPDS card no. 09-0393).

The XRD technique was employed to determine their crystalline structures and phase compositions.  Figure 2 comparably exhibits the XRD reflections of the as-obtained HBSMs, BiOBr-GC, and BiOBr-IP samples. It can be found that the solvent did not change the crystal structures of samples at all. All the distinctive reflections at 2 theta values of 10.9°, 21.9°, 25.2°, 31.7°, 32.2°, 33.1°, 39.4°, 44.7°, 46.2°, 50.7°, 56.1°, and 57.1° can be perfectly identified as the (001), (002), (101), (102), (110), (003), (112), (004), (200), (104), (114), and (212) crystal planes of the tetragonal BiOBr structure (JCPDS card no. 09-0393) with a space group of P4/nmm(129). No characteristic peaks for other impurities can be detected, indicating that phase-pure BiOBr can be obtained with the three solvent systems. Compared to the HBSMs and BiOBr-IP, the wide diffraction peaks of the BiOBr-GC imply its smaller crystal size and/or poor crystallinity, which should be associated with the high viscosity of the used GC (∼934 *μ*Pas at 20°C), much higher than that of IP (2.37 *μ*Pas). When the solvent system varies from the GC, GC/IP to IP, the viscosities of the used solvent are reduced in order. As noted, low viscosity is in favor of the higher diffusion rate of ions, generally resulting in higher supersaturation in the solution, which is greatly beneficial for the formation of nuclei [35]. Furthermore, the GC and/or IP probably serve as the soft template to direct the formation of BiOBr microstructures besides the solvent role. In the single GC system, the number of hydroxyl groups chelated on the surface of BiOBr nuclei is the largest, inhibiting the growth of the BiOBr crystal, and thus the crystal size of the obtained BiOBr-GC would be the smallest. Accordingly, the inhibiting effect of hydroxyl groups upon the growth of BiOBr crystals is reduced, accompanied by the increased IP content in the mixed solvent, thus speeding up the crystal growth of the BiOBr. In this connection, the samples with even better crystalline is prone to be observed with the solvents ranging from the GC, GC/IP to IP. Simultaneously, the crystal sizes of the as-prepared samples were also calculated according to Scherrer formula and collected in Table 1.

**Figure 3:**
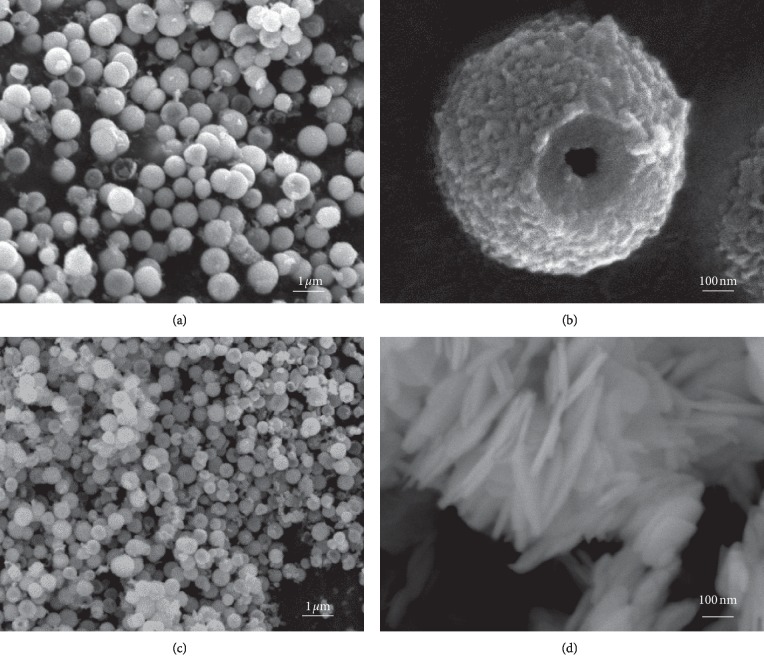
FESEM images of the (a, b) HBSMs, (c) BiOBr-GC, and (d) BiOBr-IP.

The morphology and microstructure of the BiOBr samples prepared under various conditions are clarified by FESEM analysis, as demonstrated in Figure 3.  Figure 3(a) shows the FESEM image of the HBSMs sample. Obviously, the as-prepared HBSMs product consists of a large quantity of well-dispersed submicrospheres with an average diameter of approximately 800 nm. In particular, herein, the as-resulted HBSMs with a rough surface are typically hollow, as discerned from some broken microspheres (Figure 3(b)). Inspiringly, the presence of hollow interior guarantees their larger adsorption capacity for contaminants, highlighting its potential applications in waste treatment [16–19, 36]. In the sole GC medium, the BiOBr-GC mainly displays the spherical structure with the mean diameter of ∼500 nm (Figure 3(c)), which is somewhat smaller than that of HBSMs. Additionally, the BiOBr-IP presents the flake-like structure with a lateral size of ∼500 nm and ∼30 nm in thickness (Figure 3(d)).

**Figure 4:**
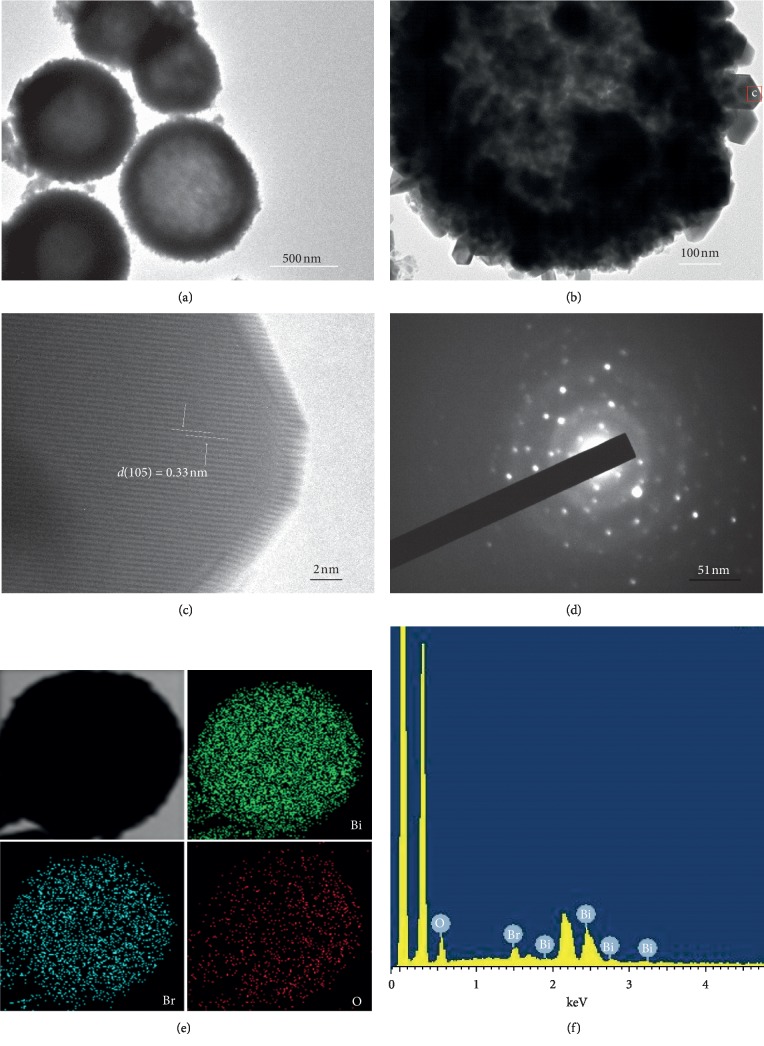
(a, b) TEM, (c) HRTEM images, (d) SAED pattern, (e) STEM and corresponding elemental (Bi, O, and Br) mapping images, and (f) EDS spectrum of the HBSMs.

The specific microstructures of the HBSMs are further elucidated by the following TEM characterizations. As observed in Figure 4(a), a relatively uniform sphere-like architecture is evident for the resultant HBSMs, which keeps high consistency with the FESEM observations above (Figures 3(a) and 3(b)). The clear contrast between the deeply dark edges and the pale center (Figure 4(a)) visually confirms the hollow feature of the HBSMs [37]. The shell of the HBSMs, which is composed of the nonclose stacking of ultrathin NSs, is estimated as ∼100 nm in thickness (Figure 4(b)). As shown in Figure 4(c), which is taken from the red rectangle region in Figure 4(b), the clear lattice fringes with a spacing of ∼0.33 nm correspond to the interplanar distance of (105) plane of the tetragonal BiOBr. Typical SAED pattern (Figure 4(d)) with a series of concentration rings and some diffraction spots reveals the polycrystalline nature of the HBSMs. Representative STEM and corresponding elemental energy dispersive spectroscopy (EDS) mapping images (Figure 4(e)) prove the uniform distributions of the Bi, O, and Br species along the HBSMs architecture. And the elemental ratio of Bi to Br is about 1 : 1 for the HBSMs, close to their stoichiometric proportion in the BiOBr (Figure 4(f)). Thanks to their hollow and non-close-packed structure, a large BET SSA of ∼55.8 m^2^·g^−1^ is obtained for the HBSMs, which is much higher than those of the BiOBr-GC (∼17.3 m^2^·g^−1^) and BiOBr-IP (∼0.94 m^2^·g^−1^), as summarized in Table 1, further indicating the tremendous influence of solvents upon the BET SSA values. The larger BET surface area and hierarchical hollow properties of the HBSMs would provide more reaction active sites and allow the separating efficiency of the photogenerated e^−^/h^+^ pairs, resulting in the enhancement in photocatalytic activities.

**Figure 5:**
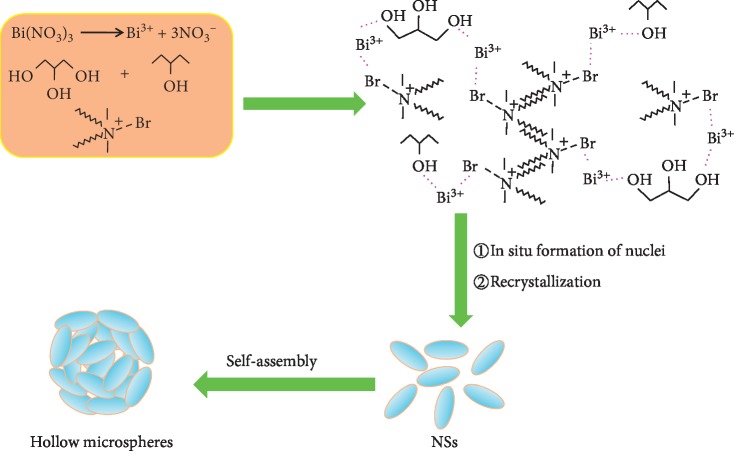
Schematic illustration of the formation process of the HBSMs.

Based on the above discussion, a plausible formation mechanism of the HBSMs can be tentatively proposed, as schematically illustrated in Figure 5. Generally, the surfactants are always used to mediate the morphology of materials [37–39]. After being dissolved in the mixed GC/IP solvent, the surfactant DDAB is self-assembled to form the special molecular structure in the mixed solvent through the hydrophobic-hydrophobic interaction. When the Bi(NO_3_)_3_·5H_2_O is subsequently added to the above mixed solvent, the hydroxyl groups of GC and IP can coordinate with Bi^3+^ and are further combined with the elemental Br in the DDAB molecules through the bridging effect of –O···Bi···Br- to form the complexes. Thus, the concentration of free Bi^3+^ ions in solution is seriously decreased, which benefits the regulation of the nucleation and growth of crystals during the solvothermal process. The solvothermal reaction environment induces the *in situ* formation of the BiOBr nuclei on the DDAB structures, and the newborn BiOBr nuclei further grow and transform into the NSs through a recrystallization process [29]. Subsequently, the formed NSs are aggregated together and self-assembled into the final HBSMs [40–42]. Further investigations are still on the way to figure out the more exact formation of the HBSMs in our lab.

**Figure 6:**
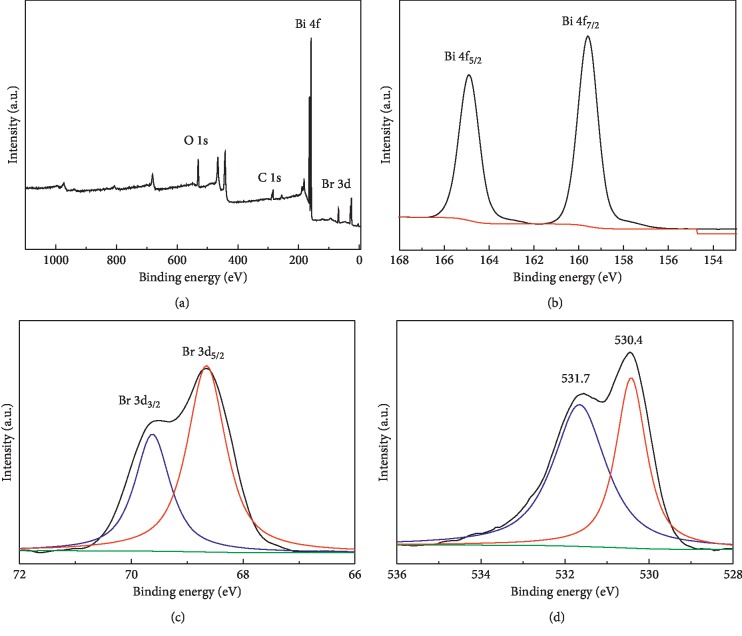
XPS spectra of the HBSMs: (a) survey, (b) Bi 4f, (c) Br 3d, and (d) O 1s.

To further investigate element compositions and chemical states of the HBSMs, XPS analysis was carried out in detail, and the corresponding results are shown in Figure 6. The XPS survey spectrum (Figure 6(a)) demonstrates that the elements of Bi, O, Br, and C coexist in the HBSMs, where the C signal (284.6 eV) should be attributed to inevitable carbon pollution [36]. In the high-resolution spectrum of Bi 4f (Figure 6(b)), two peaks at binding energies (BEs) of 159.4 and 164.7 eV can be attributed to the Bi 4f_7/2_ and Bi 4f_5/2_, which indicates the characteristic of Bi^3+^ in the HBSMs [42]. As seen from the Br 3d (Figure 6(c)), two peaks at BEs of 68.5 and 69.4 eV are assigned to Br 3d_5/2_ and Br 3d_3/2_, respectively. Meanwhile, as for the O 1s (Figure 6(d)), the peaks located at 530.4 and 531.7 eV correspond to the crystalline oxygen in the BiOBr and other excessive oxygen-containing groups (such as, ·O_2_^−^, H_2_O, etc.) on the surface of the HBSMs sample [43].

**Figure 7:**
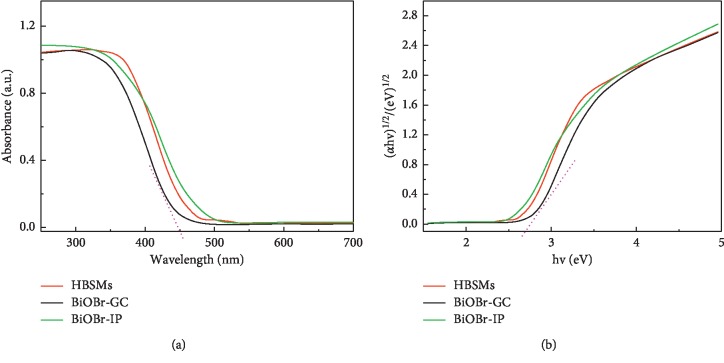
(a) UV-vis absorption spectra and (b) the plot of (Ahv)^1/2^ versus photon energy (hv) of the resultant BiOBr samples as indicated.

In general, the optical absorption property of a semiconductor, together with the migration of electrons, has close relationship with its electronic structure. Thus, it is commonly considered as an important factor to reflect the photocatalytic activities of any semiconductor [44–46]. The UV-vis diffuse-reflectance spectra of the as-prepared BiOBr samples are displayed in Figure 7(a). Remarkably, the HBSMs specimen illustrates strong photoabsorption properties in the UV light region and even visible light region, indicating its potentially high photocatalytic activities under the visible light irradiation. Compared to that of the HBSMs, the UV-vis absorption spectrum of BiOBr-GC presents a blue shift, which should be related to its relatively smaller crystal size. Besides, a proper red shift in the UV-vis absorption spectrum of the BiOBr-IP may be ascribed to quantum size effect of its nanoscale building blocks [47]. The wavelengths of absorption onset for these BiOBr samples are all collected in Table 1. According to the absorption spectra (Figure 7(a)), the plots of (Ahv)^1/2^ versus photon energy (*hv*) of the BiOBr samples are profiled in Figure 7(b). The *E*_*g*_ values can be estimated from the intercepts of the linear region in the plots of (Ahv)^1/2^ on the *Y*-axis versus photon energy (*hv*) on the *X*-axis, as plotted in Figure 7(b). The detailed *E*_*g*_ data are exhibited in Table 1. Obviously, the *E*_*g*_ decreases from 2.73 to 2.56 eV with the solvent system varying from the GC, GC/IP to IP, which suggests that all these BiOBr samples have suitable band gaps to be activated by visible light for photocatalytic degradation of organic contaminants.

### 3.2. Photocatalytic Activities

**Figure 8:**
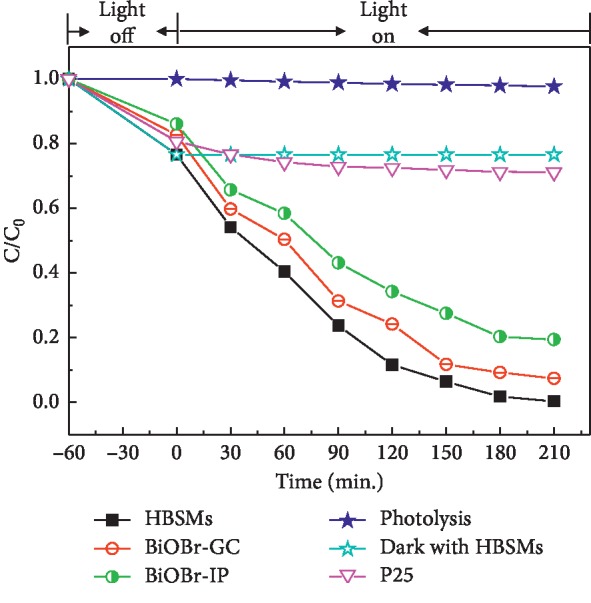
Comparable plots of the MO degradation efficiencies over the as-prepared BiOBr samples and commercial P25, direct photocatalysis of the MO under visible light irradiation, and the removal of the MO in dark with the HBSMs. C and *C*_0_ indicate the residual and initial concentrations of the MO, respectively.

The photocatalytic activities of the as-prepared BiOBr samples are purposefully evaluated by the photocatalytic degradation of the MO in water under visible light irradiation and further compared with commercial P25. The degradation efficiencies as a function of reaction time are comparatively illustrated in Figure 8. In the absence of photocatalysts, no obvious degradation of the MO is observed just under visible light irradiation. The adsorption tests were conducted in the dark, and the equilibrium adsorption study reveals that the MO concentration visually underwent slight decrease after the adsorption for 60 min for all the three photocatalysts. Corresponding dark adsorption test confirms the strongest adsorption property (∼23.4%) of the HBSMs over the MO, even stronger than the commercial P25 (∼19.3%). In spite of relatively weak adsorption capacities of the BiOBr-GC (∼17.3%) and BiOBr-IP (∼13.9%) for the MO in comparison to the P25, both of them still demonstrate higher photocatalytic efficiencies than that of the P25 upon visible light irradiation. In particular, the removal rate of the MO by the HBSMs is even close to ∼100% just after visible light irradiation for 210 min, which is much higher than those for the P25 (∼36.1%), BiOBr-GC (∼92.6%), and BiOBr-IP (∼80.6%) under the same conditions. The remarkable photocatalytic activities of the HBSMs can be attributed to the strong adsorption for the pollutant MO due to its large surface area, the high-efficiency exploitation of visible light owing to its narrower *E*_*g*_, and multiple reflections of visible light within the hollow interior, which greatly reduce the recombination of the electron-hole pairs [48, 49]. Thus, the photoinduced charges are endowed with much longer transport time and more chances to participate in the photocatalytic reactions before their recombination.

**Figure 9:**
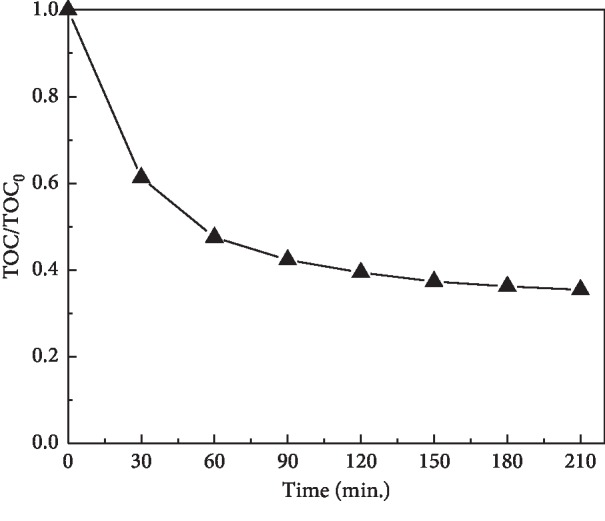
Mineralization efficiency of MO by HBSMs.

To avoid secondary pollution in the practical application, the mineralization ability of HBSMs was also evaluated by monitoring changes in TOC (Figure 9). The results validate its higher mineralisation capacity towards MO. After 210 min of visible light irradiation, the TOC removal of MO was up to 64.6%, which is slower than the decolorization rate for MO, implying that some MO molecules were actually degraded in spite of the fact that some molecules were directly mineralized to inorganic molecules [50–52].

**Figure 10:**
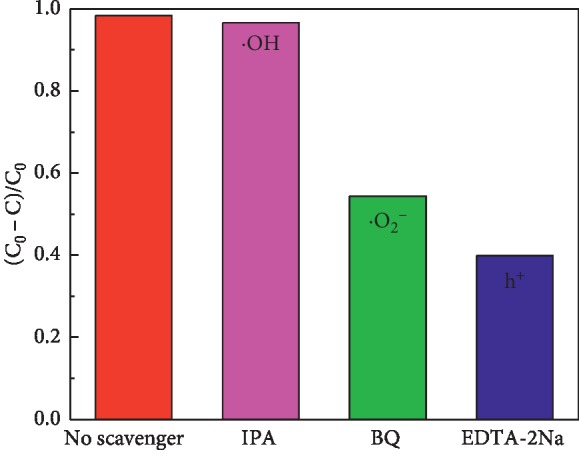
Photocatalytic degradation of MO with HBSMs with the addition of scavengers (IPA for OH, BQ for ·O_2_^−^, and EDTA-2Na for h^+^) under visible irradiation for 3 h.

Generally, some active species, such as hydroxyl radicals (·OH), superoxide radicals (·O_2_^−^), electrons (e^−^), and holes (h^+^) are generated during the photocatalytic degradation of a dye [53–56]. To explore the photocatalytic degradation mechanism of MO over HBSMs, the trapping experiments of main active species were conducted via the addition of scavengers during the photocatalytic reaction.  Figure 10 shows the photocatalytic degradation of MO over HBSMs in the absence and presence of scavengers (*i.e*., IPA for ·OH, BQ for ·O_2_^−^, and EDTA-2Na for h^+^) under visible light irradiation for 3 h. Obviously, the removal efficiency of MO decreases dramatically in the systems with the separate addition of BQ or EDTA-2Na, while it is hardly affected by adding IPA into the system, implying that the main active species in the system are ·O_2_^−^ and h^+^.

To determine the flowchart of photogenerated electron-hole pairs in HBSMs and further understand the photocatalytic reaction mechanism, the relative band positions of HBSMs were investigated. The positions of conduction band (CB) edge and valence band (VB) edge were calculated via the following empirical equations [57–59]:

(1) $$\begin{array}{c} E_{\text{CB}}=x-E_{c}-0.5E_{g},\\ E_{\text{CB}}=E_{\text{VB}}-E_{g}, \end{array}$$

where *x*, *E*_*c*_, and *E*_*g*_ are the absolute electronegativity of the photocatalyst (6.18 eV for BiOBr [59, 60]), the free electrons energy (4.50 eV), and the band gap energy, respectively. According to the equation above, the CB and VB of HBSMs can be calculated as 0.35 and 3.01 eV, respectively.

**Figure 11:**
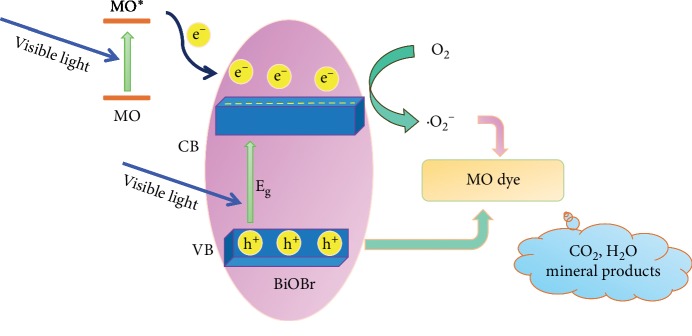
Schematic illustration for the photocatalysis mechanism of the MO over the as-obtained HBSMs.

Based on the aforementioned analysis, a feasible degradation mechanism of the MO dye by the HBSMs is tentatively proposed, as schematically described in Figure 11. Apparently, owing to the smaller band gap (2.66 eV), the electrons on the VB of HBSMs can be excited to form photoelectrons under the irradiation of visible light and then transferred to the CB of HBSMs, while the generated holes stay in the VB. As noted, the hierarchical hollow feature favors the effective yet efficient separation of the electrons and holes [16, 18]. Although VB potential of HBSMs (3.01 eV) is more positive than the redox potential of ·OH/HO^−^ (1.99 eV), the photogenerated h^+^ could hardly oxidize OH^−^ to produce ·OH, because the standard redox potential of Bi (V)/Bi (III) is only 1.59 eV [61]. Thus, these holes could not react directly with the OH^−^/H_2_O molecules to produce ·OH radicals, while MO demonstrates much lower redox potential (1.48 eV) [62], suggesting the feasibility of direct oxidation of MO by holes. Simultaneously, the CB edge potential of HBSMs (0.35 eV) was not negative enough to reduce O_2_ to produce ·O_2_^−^ due to the standard redox potential of O_2_/·O_2_^−^ (−0.046 eV) [63, 64]; thus the e^−^ in the CB of HBSMs could not reduce the adsorpted O_2_ to generate ·O_2_^−^. However, the fact that ·O_2_^−^ is the main active substance is not in accordance with the abovementioned trapping experiment. The reason may be related to visible light with more energy (*λ* > 400 nm, energy less than 3.1 eV); the photoelectrons may be excited to a higher level of CB (−0.09 eV). Meanwhile, the MO absorbs the incident photo flux due to the photosensitization phenomenon arising from the transition of MO to MO^∗^. The collected electrons in the higher energy level of CB can be scavenged by the absorbed molecular oxygen on its surface to produce more ·O_2_^−^ owing to the more negative potential (−0.09 eV) than that of ·O_2_^−^ formation potential (−0.046 eV). Thus, such synergistic effect contributed by electrons in the CB and holes in the VB of the HBSMs effectively ensures that the MO is fully oxidized to the CO_2_, H_2_O, and some mineral products. As a result, the HBSMs demonstrate striking photocatalytic degradation of the dye MO under visible light irradiation.

**Figure 12:**
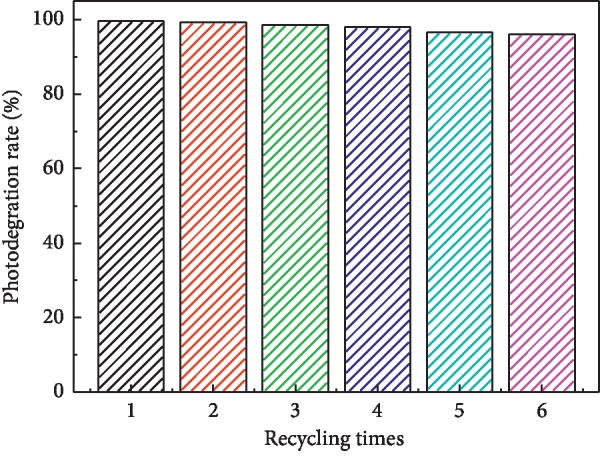
Cycling stability of the HBSMs for degradation of the MO.

**Figure 13:**
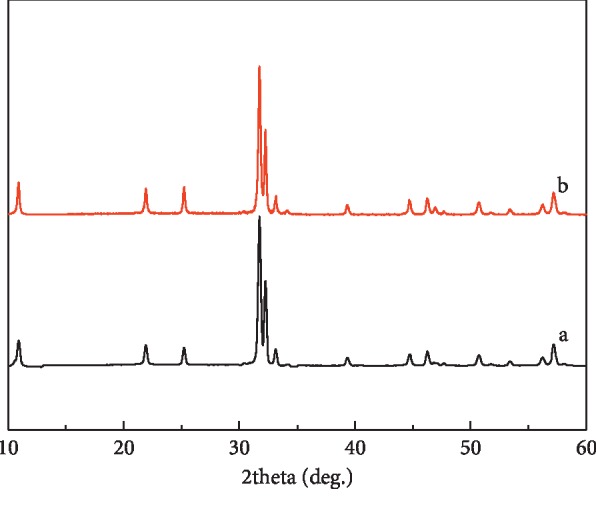
XRD spectra of HBSMs (a) and HBSMs after six cycles (b) for the photocatalytic degradation of the MO.

The excellent reusability of any photocatalyst is also recognized as a key factor for its practical applications. To evaluate the photocatalytic stability of the HBSMs, photocatalytic activities of the HBSMs are investigated by circulating runs in the degradation of the MO under visible light radiation. As can be inferred from Figure 12, the shrinking of degradation rate is still kept within ∼4% even up to six consecutive cycles under the same photocatalytic conditions. In addition, the XRD pattern of HBSMs after six cycles for the photocatalytic degradation of the MO presented no obvious change in comparison with its original pattern (Figure 13), which further confirms the remarkable stability of the resultant HBSMs. The observation here implies that the HBSMs own superior cycling stability, and no obvious photocorrosion takes place during the photocatalytic degradation of the MO molecules, which is of particular significance for its commercial applications.

## 4. Conclusions

In summary, the hierarchical hollow BiOBr submicrospheres were successfully prepared *via* a facile and efficient solvothermal strategy in a mixed solvent of GC and IP. The key role of the DDAB in the formation of the HBSMs and detailed effects of various solvents on the crystallinity, morphology, and microstructure of the BiOBr products were investigated. Accordingly, the formation mechanism of the HBSMs was rationally proposed here. More strikingly, the obtained HBSMs demonstrated excellent photocatalytic activity for efficient photodegradation of the organic MO and possessed good reusability under the visible light radiation, indicating their promising appealing application in waste water treatment. Furthermore, the synthetic methodology presented here can be extended to other hierarchical hollow materials with versatile applications.

## Figures and Tables

**Table 1:** Summary of detailed parameters of control experiments and corresponding structural data of the HBSMs, BiOBr-GC, and BiOBr-IP products.

Samples	Reaction medium	Shape and crystal size (nm)	Band edge (nm)	*E*_*g*_ (eV)	*S*_BET_ (m^2^·g^−1^)
HBSMs	20 mL GC + 20 mL IP	Spheres (13.49)	467	2.66	∼55.8
BiOBr-GC	40 mL GC	Spheres (7.23)	454	2.73	∼17.3
BiOBr-IP	40 mL IP	Sheets (14.52)	485	2.56	∼0.94

## Data Availability

The data used to support the findings of this study are available in Table 1 and Figures 1–13 of this article.
